# Identifying heterotic groups and testers for hybrid development in early maturing yellow maize (*Zea mays*) for sub‐Saharan Africa

**DOI:** 10.1111/pbr.12822

**Published:** 2020-04-20

**Authors:** Benjamin Annor, Baffour Badu‐Apraku, Daniel Nyadanu, Richard Akromah, Morakinyo A. B. Fakorede

**Affiliations:** ^1^ CSIR‐Crops Research Institute Kumasi Ghana; ^2^ Department of Crop and Soil Sciences Faculty of Agriculture Kwame Nkrumah University of Science and Technology Kumasi Ghana; ^3^ International Institute of Tropical Agriculture (UK) Limited Croydon UK; ^4^ Cocoa Research Institute of Ghana AkimTafo Ghana; ^5^ Obafemi Awolowo University Ile‐Ife Nigeria

**Keywords:** combining ability, drought, low soil nitrogen, stress tolerance, *Striga hermonthica*, testcrosses, *Zea mays* L

## Abstract

Identification of heterotic groups and efficient testers, which are important prerequisites for the development of outstanding hybrids, has been a major challenge to its success, especially for early and extra‐early germplasm. This study was carried out to (a) identify the most efficient heterotic grouping method for classifying a set of inbred lines and (b) determine the efficiency of testers in classifying inbred lines into heterotic groups. A total of 205 hybrids obtained by crossing 41 inbred lines with five standard testers were evaluated together with five hybrid checks under drought, low soil nitrogen (N), *Striga*‐infested and optimal environments in Nigeria between 2014 and 2016. The heterotic group's specific and general combining ability (HSGCA) method was more effective in classifying the inbred lines into heterotic groups. Testers TZEI 17 and TZEI 23 were the most efficient across environments and could be invaluable for classifying other lines into heterotic groups and assessing combining ability of maize inbreds. In addition, these testers and heterotic groups represent an invaluable resource for development of outstanding hybrids in sub‐Saharan Africa (SSA).

## 1 INTRODUCTION

Maize (*Zea mays*) is a major source of food for human consumption and livestock production. It is also used as a raw material for many agro‐allied industries in the world (Undie, Uwah, & Attoe, 2012). In West Africa (WA), maize production has been increasing steadily over the years due to its wide adaptation and uses. However, its production is characterized by low grain yield (1.8 t/ha) compared with the yields obtained in other parts of the world (FAOSTAT 2008, 2012, 2016; Sserumaga et al., 2014). This situation is partly due to the non‐availability of high‐yielding hybrids with tolerance to drought and low nitrogen (low N) in the soil, and resistance to infestation of *Striga hermonthica* (Del.) Benth. Hybrids revolutionized maize production in developed countries during 1930–1950 but have not been widely adopted in most sub‐Saharan African (SSA) countries. Adoption of hybrid maize by farmers in many developing countries has been very slow, although it has now been increasing gradually in SSA.

An important requirement for the development of high‐yielding commercial hybrids is the availability of information on the heterotic groups and patterns of the available inbred lines in a breeding programme (Barata & Carena, 2006; Fan et al., 2018). Globally, inbred lines and the derived commercial hybrids resulting from them must be stress tolerant. Stresses common to all SSA countries include drought and low N and infestation by parasitic weeds such as *Striga hermonthica*, especially in WCA. Classification of inbred lines into heterotic groups under the different environmental conditions has been initiated by several researchers in SSA (Agbaje, Badu‐Apraku, & Fakorede, 2008; Badu‐Apraku, Fakorede, et al., 2015; Badu‐Apraku et al., 2013; Menkir, Badu‐Apraku, Thé, & Adepoju, 2003) but limited success has been achieved. Taking a cue from the experience and the standards attained over a long time by the USA maize breeders, heterotic grouping of inbred lines has to be a continuous exercise in maize breeding programmes. Heterotic groups and testers for the newly developed or introduced set of inbred lines in a breeding program must be determined (Barata & Carena, 2006; Fan et al., 2018; Fan, Tan, Yang, & Chen, 2004). Additionally, it is important for any successful or effective breeding programme aimed at developing outstanding drought, low N and *Striga* resistant hybrids to assess the heterotic groups under individual stress, non‐stress (optimum), and across environments.

The International Institute of Tropical Agriculture (IITA), Ibadan‐Nigeria, in collaboration with the International Maize and Wheat Improvement Center (CIMMYT) in Kenya and with national programmes, has for years been developing and releasing OPVs in all maturity groups and hybrids in the late and intermediate groups but it is only recently that a few stress‐tolerant/resistant early and extra‐early hybrids have been developed and released in some WCA countries. However, maize researchers have consistently observed that the performance of early (90–95 days to physiological maturity) and extra‐early (80–85 days to physiological maturity) yellow maize OPVs and hybrids lag grossly behind their white counterparts in SSA because for a long time research emphasis on maize had been on the white endosperm maize. However, recently, there has been increased research emphasis on the use of yellow endosperm maize because of the high demand for the poultry industry and for human consumption to address vitamin A deficiencies. The yellow maize is preferred for poultry feed because it imparts the yellow colour to the egg yolk and contributes to human nutrition. Therefore, it is imperative to identify the heterotic groups of the newly developed or introduced early maturing yellow maize inbred lines so that they could be successfully used for the development of high‐yielding hybrids and synthetic varieties for commercialization in SSA. Hybrid maize varieties are preferred by progressive farmers in SSA due to the high yield compared to OPVs (Ayinde, Fola, & Ibrahim, 2011; Correjado & Magulama, 2008) and uniformity in growth and other agronomically desirable characteristics.

Results of the attempts by maize breeders to identify the most efficient heterotic grouping methods have not been consistent. For example, Fan, Miles, Takahashi, and Yao (2009) and Badu‐Apraku, Fakorede, et al. (2015) compared the specific combining ability (SCA) of several lines using molecular markers and the heterotic group's specific and general combining ability (HSGCA) methods and found the HSGCA method to be the most efficient based on the breeding efficiency (the average of the proportion of total inter‐heterotic group hybrids that is due to superior high‐yielding inter‐heterotic group hybrids plus the proportion of total low‐yielding intra‐heterotic group hybrids that is due to the low‐yielding intra‐heterotic group hybrids). In contrast, Badu‐Apraku, Annor, et al. (2015), Badu‐Apraku, Fakorede, Talabi, et al. (2016) reported the heterotic grouping based on molecular markers as the most efficient in a study involving early maturing quality protein maize (QPM) inbred lines. The contrasting results reported have been attributed to the differences in the genetic materials used (Badu‐Apraku, Fakorede, Gedil, et al., 2016). It is therefore of utmost importance to classify the newly developed early maturing maize inbred lines using the most efficient grouping method to identify the best set of new inbred lines for effective use in maize breeding programmes.

Another important requirement of any successful maize breeding programme is the availability of efficient testers, which could effectively discriminate and classify inbred lines into appropriate heterotic groups for the development of high‐yielding hybrids and synthetic varieties. An effective tester should be able to rank inbred lines correctly for performance in hybrid combinations and increase the differences between testcrosses for efficient discrimination (Rawlings & Thompson, 1962). Several early maturing yellow endosperm inbred lines [TZEI 10 (A), TZEI 17 (B), TZE 23 (C), TZEI 129 (D) and ENT 13 (E)] have been identified as standard testers in the IITA Maize Improvement Program (MIP). It is therefore important to authenticate the efficiency of these early inbred lines as testers which could be effectively utilized for grouping other inbred lines and for the development of productive hybrids and synthetic varieties. The objectives of the present study were to (a) identify the most efficient of the two heterotic grouping methods, HSGCA, and genetic distance from SNP markers for SSA agro‐ecological conditions and (b) determine the efficiency of testers in classifying selected tropical inbred lines into heterotic groups.

## 2 MATERIALS AND METHODS

### 2.1 Experimental materials and generation of testcrosses

Forty‐one S_7_ inbred lines of maize (*Zea mays* L.) developed in the IITA, and CIMMYT maize improvement programmes (MIP) were used in the present study. Thirty‐one of the lines were inbreds developed from TZEI 11 x TZEI 8, with five from TZE‐Y Pop STR Co, three from TZE Comp5‐Y C6, one from M37W/ZM607#bF37sr‐2‐3sr‐6‐2‐X and one from 87036/87923 populations. The inbred lines were selected based on the contrasting responses to drought and *Striga* infestation (Table 1). The five early maturing yellow endosperm elite inbreds (three of the testers were identified from a set of inbreds derived from TZE‐Y Pop STR Co, one from TZE Comp5‐Y, and one from M37W/ZM607#bF37sr‐2‐3sr‐6‐2‐X populations) testers identified in the IITA MIP were crossed to the 41 inbred lines using the line by tester mating design during the dry season of 2014 in Ibadan, Nigeria, to obtain 205 testcross hybrids. The 205 hybrids plus five drought, low N and *Striga* resistant hybrid checks were evaluated under four research conditions (drought, low N, *Striga*‐infested and optimal conditions) in Nigeria from 2014 to 2016 (Table 1).

**Table 1:** Description of early maturing maize inbred lines used in the study

S/*N*	Inbred	Designation	Source	Reaction to drought	Reaction to *Striga*
1	TZEI 415	(TZEI 11 × TZEI 8) S_7_ inb 18‐1/3‐1/2‐1/1	IITA	Tolerant	Tolerant
2	TZEI 428	(TZEI 11 × TZEI 8) S_7_ inb 30‐3/3‐7/9‐1/1	IITA	Tolerant	Tolerant
3	TZEI 430	(TZEI 11 × TZEI 8) S_7_ inb 37‐1/3‐3/3‐1/1	IITA	Tolerant	Susceptible
4	TZEI 432	(TZEI 11 × TZEI 8) S_7_ inb 47‐3/4‐4/11‐1/1	IITA	Tolerant	Susceptible
5	TZEI 433	(TZEI 11 × TZEI 8) S_7_ inb 81‐1/4‐8/10‐1/1	IITA	Tolerant	Susceptible
6	TZEI 439	(TZEI 11 × TZEI 8) S_7_ inb 92‐2/5‐3/7‐1/1	IITA	Tolerant	Susceptible
7	TZEI 441	(TZEI 11 × TZEI 8) S_7_ inb 92‐5/5‐4/5‐1/1	IITA	Tolerant	Susceptible
8	TZEI 442	(TZEI 11 × TZEI 8) S_7_ inb 92‐5/5‐5/5‐1/1	IITA	Tolerant	Susceptible
9	TZEI 443	(TZEI 11 × TZEI 8) S_7_inb 95‐2/2‐1/5‐1/1	IITA	Tolerant	Tolerant
10	TZEI 449	(TZEI 11 × TZEI 8) S_7_inb 107‐6/6‐4/7‐1/1	IITA	Tolerant	Tolerant
11	TZEI 450	(TZEI 11 × TZEI 8) S_7_ inb 112‐2/4‐4/4‐1/1	IITA	Tolerant	Tolerant
12	TZEI 455	(TZEI 11 × TZEI 8) S_7_ inb 133‐3/3‐3/3‐1/1	IITA	Tolerant	Susceptible
13	TZEI 461	(TZEI 11 × TZEI 8) S_7_ inb 148‐1/3‐4/6‐1/1	IITA	Tolerant	Susceptible
14	TZEI 462	(TZEI 11 × TZEI 8) S_7_ inb 148‐1/3‐6/6‐1/1	IITA	Tolerant	Tolerant
15	TZEI 464	(TZEI 11 × TZEI 8) S_7_ inb 148‐3/3‐3/5‐1/1	IITA	Tolerant	Susceptible
16	TZEI 465	(TZEI 11 × TZEI 8) S_7_ inb 148‐3/3‐4/5‐1/1	IITA	Tolerant	Tolerant
17	TZEI 470	(TZEI 11 × TZEI 8) S_7_ inb 154‐3/3‐1/5‐1/1	IITA	Tolerant	Tolerant
18	TZEI 472	(TZEI 11 × TZEI 8) S_7_ inb 154‐3/3‐4/5‐1/1	IITA	Tolerant	Tolerant
19	TZEI 474	(TZEI 11 × TZEI 8) S_7_ inb 170‐1/3‐2/6‐1/1	IITA	Tolerant	Tolerant
20	TZEI 483	(TZEI 11 × TZEI 8) S_7_ inb 184‐3/3‐4/6‐1/1	IITA	Tolerant	Tolerant
21	TZEI 484	(TZEI 11 × TZEI 8) S_7_ inb 184‐3/3‐6/6‐1/1	IITA	Tolerant	Tolerant
22	TZEI 486	(TZEI 11 × TZEI 8) S_7_ inb 185‐1/2‐4/5‐1/1	IITA	Tolerant	Tolerant
23	TZEI 494	(TZEI 11 × TZEI 8) S_7_inb 201‐1/2‐3/6‐1/1	IITA	Susceptible	Tolerant
24	TZEI 495	(TZEI 11 × TZEI 8) S_7_ inb 201‐1/2‐4/6‐1/1	IITA	Tolerant	Tolerant
25	TZEI 507	(TZEI 11 × TZEI 8) S_7_ inb 238‐3/4‐5/6‐1/1	IITA	Tolerant	Tolerant
26	TZEI 508	(TZEI 11 × TZEI 8) S_7_ inb 248‐1/4‐1/4‐1/1	IITA	Tolerant	Susceptible
27	TZEI 515	(TZEI 11 × TZEI 8) S_7_ inb 258‐1/4‐2/6‐1/1	IITA	Tolerant	Susceptible
28	TZEI 516	(TZEI 11 × TZEI 8) S_7_ inb 258‐1/4‐5/6‐1/1	IITA	Tolerant	Tolerant
29	TZEI 518	(TZEI 11 × TZEI 8) S_7_ inb 258‐2/4‐5/7‐1/1	IITA	Tolerant	Tolerant
30	TZEI 520	(TZEI 11 × TZEI 8) S_7_ inb 263‐1/2‐2/6‐1/1	IITA	Tolerant	Tolerant
31	TZEI 522	(TZEI 11 × TZEI 8) S_7_ inb 282‐2/3‐1/5‐1/1	IITA	Tolerant	Tolerant
32	TZEI 124	TZE‐Y Pop STR Co S_7_ Inbred 3‐1‐3	IITA	Susceptible	Tolerant
33	TZEI 16	TZE Comp5‐Y C6 S_7_ Inbred 31	IITA	Tolerant	Susceptible
34	TZEI 24	TZE‐Y Pop STR Co S_7_ Inbred 142‐2‐2	IITA	Tolerant	Resistant
35	TZEI 160	TZE‐Y Pop STR Co S_7_ Inbred 102‐2‐3	IITA	Tolerant	Tolerant
36	TZEI 161	TZE‐Y Pop STR Co S_7_ Inbred 103‐2‐3	IITA	Tolerant	Tolerant
37	TZEI 173	TZE Comp5‐Y C6 S_7_ Inbred 21A	IITA	Susceptible	Tolerant
38	TZEI 175	TZE Comp5‐Y C6 S_7_ Inbred 25B	IITA	Susceptible	Tolerant
39	TZEI 182	TZE‐Y Pop STR Co S_7_ Inbred 152‐2‐2	IITA	Tolerant	Tolerant
40	ENT 8	[M37W/ZM607#bF37sr‐2‐3sr‐6‐2‐X]‐8‐2‐X‐1‐BB	CIMMYT	Tolerant	Susceptible
41	ENT 17	[(87036/87923)‐X‐800‐3‐1‐X‐1‐B‐B‐1‐1‐1‐B‐B‐xP	CIMMYT	Tolerant	Tolerant
42	TZEI 23 (C)	TZE‐Y Pop STR Co‐ Inbred 62‐2‐3	IITA	Tolerant	Resistant
43	TZEI 129 (D)	TZE‐Y Pop STR Co‐Inbred 16‐1‐3	IITA	Tolerant	Susceptible
44	TZEI 10(A)	TZE‐Y Pop STR Co‐ Inbred 152	IITA	Tolerant	Tolerant
45	TZEI 17 (B)	TZE Comp5‐Y C6‐ Inbred 35	IITA	Tolerant	Tolerant
46	ENT 13 (E)	[M37W/ZM607#bF37sr‐2‐3sr‐6‐2‐X]‐8‐2‐X‐1‐BB‐	CIMMYT	Tolerant	Susceptible

Note

(TZEI 11 × TZEI 8) S_7_ inb 18‐1/3‐1/2‐1/1 = TZEI 415 was developed from a cross between TZEI 11 × TZEI 8, taken through seven cycles of inbreeding (S_7_) and was the 18th line selected after the first cycle (S_1_) of inbreeding. This was followed by several cycles of repeated selfing and selection at the different stages of inbreeding.

### 2.2 Field experiments

The 205 hybrids plus five checks were evaluated under terminal drought (where planting was done in such a way that flowering and grain filling periods coincided with the natural drought) in a drought‐prone location, Bagauda (lat. 12°11'N, long. 8°37'E, with elevation of 580 masl and 800 mm annual rainfall) during the 2016 rainy season. The evaluation of the hybrids was also conducted under induced drought stress at Minjibir (lat. 12°00'N, long. 8°22'E, with elevation of 445 masl and 800 mm annual rainfall) and Ikenne (6°53''N, 30°42'E, 60 masl, 1,200 mm annual rainfall) during the 2014/2015 and 2015/2016 dry seasons, respectively (Table S1). Drought stress at both locations was induced by suspending irrigation at 28 days after planting (DAP); however, at Minjibir irrigation was resumed after flowering due to the high intensity of the heat during the plant growth and development period. A sprinkler irrigation system which supplied 17 mm of water weekly was used. Fertilizer was applied as NPK (15‐15‐15) at planting at the rate of 60 kg NPK/ha and top‐dressed with urea at 60 kg N/ha at two weeks after planting (WAP). Under the terminal drought, basal and top dressing were carried out at two and four WAP.

The hybrids were also evaluated under low N (30 kg/ha according to soil tests) conditions at Mokwa (9^o^18'N, 5°4'E, 457 masl, 1,100 mm annual rainfall) during the 2015 and 2016 rainy seasons and Ile‐Ife (7°28'N, 4°33'E, and 244 masl, 1,200 mm annual rainfall) during the 2015 rainy season (Table S1). The low N fields at both locations were depleted of N by growing maize continuously at high density for several years and removing the biomass after each harvest. Soil samples taken from 0 to 15 cm depth before field preparation were analysed for nitrogen (N), phosphorus (P) and potassium (K) contents at the IITA analytical services laboratory, Ibadan, Nigeria, following the Kjeldahl digestion and colorimetric methods (Bremner & Mulvaney, 1982). The Mokwa soil contained 0.033% of N, 4.11 mg/kg of P and 0.14 cmol/kg of K while that of Ile‐Ife had 0.081% of N, 4.04 mg/kg of P and 0.23 cmol/kg of K. Nitrogen fertilizer (Urea) was applied at two WAP following thinning to bring the total available N to 30 kg/ha as indicated by the soil tests. Single superphosphate and muriate of potash fertilizers were applied to obtain 60 kg/ha each of P and K.

Also, the hybrids were evaluated under optimal growing conditions during the 2015 and 2016 rainy seasons at Mokwa, Ikenne, Abuja (9°16'N, 7°20'E, 300 m asl, 1,500 mm annual rainfall) and during the 2015 rainy season at Ile‐Ife (Table S1). The compound fertilizer, NPK (15:15:15), was applied to all the optimal trials at two WAP to provide 60 kg/ha each of N, P and K and top‐dressed at four WAP with 60 kg N/ha. The low N, drought and optimal fields were kept weed‐free by the application of atrazine and gramozone as preemergence and postemergence herbicides at 5 L/ha each of primextra and paraquat and later by manual weeding.

The hybrids were evaluated under artificial *Striga* infestation at Mokwa and Abuja in 2015 and 2016 (Table S1). Ethylene gas was injected into the soil at two weeks before planting to stimulate suicidal germination of existing *Striga* seeds. The infestation with *Striga* was carried out using the method of Kim (1991). The *Striga hermonthica* seeds used were collected from sorghum fields in the preceding season, stored for at least six months and mixed with finely sieved sand in the ratio of 1 : 99 by weight. A standard scoop calibrated to ensure that about 5,000 germinable *Striga* seeds were placed in each planting hole was used for the artificial infestations. To ensure good germination of the *Striga* seeds and attachment of *Striga* plants to the roots of the maize plants, fertilizer application was delayed until 21 DAP when 30 kg/ha each of N, P and K was applied as NPK 15–15–15. Weeds other than *Striga* were controlled by hand weeding.

A 14 × 15 alpha‐lattice design with two replications was used in all experiments conducted under the contrasting environments. The experimental units were single‐row plots, 3 m long each with row and hill spacing of 0.75 and 0.40 m. Three seeds were sown per hill and later the seedlings were thinned to two at two WAP to obtain a population density of about 66,666 plants/ha.

### 2.3 Single nucleotide polymorphism marker assays

#### 2.3.1 DNA extraction

Seeds of 41 early maturing maize inbred lines and the five lines used as testers were planted at IITA, Ibadan, Nigeria. Two weeks after planting, leaf samples were collected from 30 seedlings of each inbred in the field. The leaf samples were then bulked, lyophilized, carefully packaged, labelled and transferred to CIMMYT Mexico for DNA extraction and genotyping. DNA was extracted at the CIMMYT Mexico Bioscience laboratory using the CIMMYT protocol (http://www.generationcp.org/capcorner/chile_wksp_2005/manuals/manual_01.pdf).

#### 2.3.2 Genotyping by sequencing

The enzyme ApeKI was used for digestion and creating a genotyping by sequencing (GBS) library with unique barcodes for each inbred line as described by Elshire et al. (2011). The reads obtained from the GBS library were called in the GBS pipeline Tassel version 3.0.147 which is an extension to the Java program TASSEL (Bradbury et al., 2007). The sequences were aligned to the maize reference genome B73 RefGen v1 after filtering using the Burrows–Wheeler alignment tool (BWA) (Schnable, Ware, Fulton, Stein, & Wei, 2009). The procedure produced 51,009 SNPs covering all the ten chromosomes of the maize genome, out of which 3,508 SNP loci, having a minor allele frequency of more than 5% and no missing values, were selected using TASSEL version 5.0, and employed for analyzing the genetic diversity of the inbred lines in the present study. The pair‐wise Rogers (1972) genetic distances were estimated among the inbred lines using PowerMarker version 3.25 (Liu & Muse, 2005).

#### 2.3.3 Field phenotyping

Data were recorded on all plots for days to silking, and days to anthesis, anthesis–silking interval (ASI), plant and ear heights, root lodging, stalk lodging, ear aspect, ear rot and ears per plant. In addition, plant aspect was recorded on the drought, low N and optimal plots, stay‐green characteristic on the low N and drought plots as described by Badu‐Apraku, Fakorede, Gedil, et al. (2016) and *Striga* damage and number of emerged *Striga* plants on only the *Striga*‐infested plots. Under optimal and *Striga‐*infested environments, a shelling percentage of 80% was assumed for each plot. Grain yield was obtained from the ear weight and converted to kg/ha by adjusting the moisture content to 15%. For experiments conducted under low N and drought conditions, harvested ears from each plot were shelled to determine the percentage grain moisture. Grain yield in kg/ha adjusted to 15% moisture content was then computed from the shelled grain weight. The 80% shelling percentage was assumed for entries only under *Striga* infestation and optimal conditions because grain filling is usually normal under such conditions.

### 2.4 Statistical analysis

Analysis of variance (ANOVA) was performed across environments (location–year combinations) with PROC GLM in the Statistical Analysis System (SAS) using a random statement with test option (SAS Institute, 2011). In the combined ANOVA, genotypes were considered as a fixed factor while environment, replication within environment and incomplete blocks within replication by environment were regarded as random factors.

A line x tester analysis of variance was used to determine the statistical significance of GCA‐line, GCA‐tester, SCA‐hybrid and their interactions with the environments as described by Amegbor, Badu‐Apraku, and Annor (2017). Of particular importance in this study was the SCA x environment interaction, an indication of possible differences in the heterotic grouping of the lines under the different environmental conditions.

Heterotic grouping of the inbred lines across low N, drought, *Striga*‐infested and optimal growing environments was performed using the HSGCA [Mean of Hybrid(ij) ‐ Mean of Tester(i)] of grain yield proposed by Fan et al. (2009), genetic distance of maize inbred lines from SNP‐based molecular marker method as detailed by Barata and Carena (2006), Li, Yuan, Li, Zhang, and Li (2003), Menkir, Melake‐Berhan, The, Ingelbrecht, and Adepoju (2004) and Badu‐Apraku, Fakorede, Talabi, et al. (2016).

The efficiencies of the two heterotic grouping methods were compared by arranging the 205 testcrosses from the highest to the lowest based on the means of grain yield across environments. The total number of hybrids for each method was divided into two major groups: intergroup and intragroup crosses. These two groups were subsequently divided into high‐yielding hybrids (Yield Group 1 with a mean grain yield ranking among the top 68 hybrids), intermediate‐yielding hybrids (Yield Group 2 with a grain yield between the 69th and 136th hybrid) and low‐yielding hybrids (Yield Group 3 with a mean grain yield between 137th and 205th hybrid). The best classification method was identified based on the breeding efficiency proposed by Fan et al. (2009) and modified by Badu‐Apraku, Fakorede, Talabi, et al. (2016). The equation for estimating the breeding efficiency is as shown below:

$$\text{Breeding efficiency}\;=\;\frac{\left[\frac{\text{HY INTERGH}}{\text{TN INTERGH}}\times 100\right]+\left[\frac{\text{LY INTERGH}}{\text{TN INTERGH}}\times 100\right]\;}{2}$$

where HY INTERGH = number of high‐yielding inter‐heterotic group hybrids,

TN INTERGH = total number of inter‐heterotic group hybrids,

LY INTRAGH = number of low‐yielding intra‐heterotic group hybrids,

TN INTRAGH = total number of intra‐heterotic group hybrids.

To identify the most efficient inbred tester across the four contrasting research conditions, data on grain yield mean values across the four research conditions adjusted for block and replication effects were subjected to genotype main effect plus genotype x environment interaction (GGE) biplot analysis (testers were used in the analysis in place of environments) as described by Yan and Hunt (2002).

## 3 RESULTS

Analysis of variance of grain yield and other traits across contrasting environments.

Significant (*p* < .05) mean squares of environments (E), hybrid (G) and hybrid × environment interactions (GEI) were observed for grain yield and most measured agronomic traits across environments (Table 2). Partitioning of the hybrid components of variation into GCA of line (GCA‐line) and GCA of tester (GCA‐tester) and SCA mean squares exhibited significant gains for GCA‐line, GCA‐tester and SCA for grain yield and most measured agronomic traits across environments. The GCA‐line × E and GCA‐tester x E interaction mean squares were also significant for most measured traits (Table 2). In contrast, the mean squares of the SCA × E interactions were not significantly different for most measured traits (Table 2).

**Table 2:** Mean squares of grain yield and other agronomic traits of early maturing maize hybrids and five checks across 17 contrasting environments in Nigeria between 2014 and 2016

Source	DF	YIELD	POLLEN	DYSK	ASI	PLHT	EHT	EASP	EROT	EPP	DF	PASP	DF	STGR
Env	16	848,804**	1862.51**	3,625.96**	411.37**	182,027**	78,014**	37.50**	2,996.61**	2.53**	12	92.43**	5	506.67**
Rep(Env)	17	12,982**	62.80**	87.29**	6.92**	2,632**	2,121**	4.48**	128.39**	0.13**	13	4.86**	6	25.77**
Block(Env x Rep)	442	2,245**	5.75**	8.23**	1.58**	639**	266**	1.07**	8.68**	0.03**	338	1.12**	156	1.88**
Hybrid	209	12,034**	62.47**	83.04**	4.74**	3,120**	788**	4.17**	16.73**	0.05**	209	4.87**	209	1.77**
GCA‐line	40	19,327**	208.43**	306.88**	15.97**	3,077**	824**	4.66**	47.31**	0.09**	40	6.71**	40	3.05**
GCA‐tester	4	188,137**	880.46**	956.38**	24.76**	116,079**	27,107**	79.47**	118.94**	0.39**	4	117.24**	4	14.61**
SCA	160	6,079**	13.34**	15.58**	1.95**	772**	234**	2.30**	8.27**	0.04**	160	1.83**	160	1.36^*^
Env x Hybrid	3,344	1,459**	4.95**	6.75**	1.57**	398**	150**	0.65**	5.50**	0.03**	2,508	0.62**	1,045	1.04**
En x GCA‐line	640	2,365**	10.88**	15.47**	2.14**	462**	207**	1.01**	7.77**	0.04**	480	0.84**	200	1.40**
Env x GCA‐ tester	64	9,846**	12.76**	20.83**	5.95**	3,642**	917**	3.68**	27.10**	0.12**	48	2.24**	20	4.31**
Env x SCA	2,560	1,265**	4.33**	5.43**	1.48**	356**	143**	0.60^*^	5.24^*^	0.03**	1920	0.61^*^	800	1.09
Error	3,484	986	2.86	4.33	1.27	287	131	0.56	4.85	0.02	2,664	0.55	1,229	1.10

*,** = Significant at 0.05 and 0.01 probability levels, respectively; ASI, anthesis–silking interval; DYSK, days to 50% silking; EASP, ear aspect; EHT, ear height (cm); Env, environment; EPP = ears per plant; EROT, ear rot; PASP, plant aspect; PLHT, plant height (cm); Pollen = days to 50% anthesis; Rep, replication; STGR = stay‐green characteristic. The error terms should only be used to test the last source of variation; YIELD, grain yield (t/ha).

### 3.1 Efficiency of testers based on discriminating ability across environments

**Figure 1:**
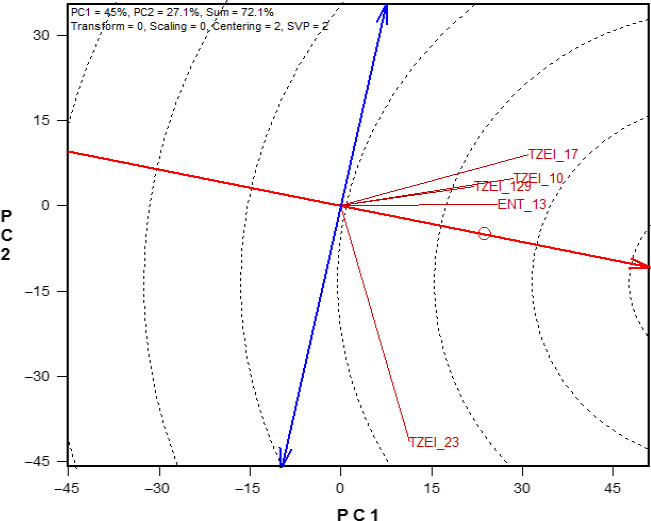
A vector view of genotype main effect plus genotype by environment (GGE) biplot showing the discriminating power and representativeness of the testers across drought, low soil nitrogen, *Striga*‐infested and optimal environments in Nigeria between 2014 and 2016 [Colour figure can be viewed at wileyonlinelibrary.com]

The efficiency of a tester was assessed by the average environment (tester) coordination (AEC) view of the GGE biplot (Figure 1) as described by Yan (2014) and Akinwale, Badu‐Apraku, Fakorede, and Vroh‐Bi (2014). The thick single‐arrow line is referred to as the average environment (tester) coordinate abscissa (AEC abscissa) while the double‐headed arrow line is called the AEC ordinate. The efficiency is determined by the relationship among the testers and the length of the tester vector. In the biplot display, the cosine of the angle between any two tester vectors indicates the correlation coefficient between the testers. The smaller the angle between any two testers, the more closely related the testers are in classifying inbred lines into heterotic groups. In addition, in the biplot display, the rays connecting the tester label to the biplot origin are described as tester vectors and the vector length of a tester approximates the standard deviation, which measures the magnitude (discriminating power) of its ability to assess the grain yield of the crosses. Testers with shorter vectors provide little or no information about the entries evaluated compared to those with longer vectors. Based on these criteria, the ranking based on discriminating ability of the testers was as follows: TZE 23 (C)> TZEI 17 (B)> TZEI 10 (A)> ENT 13 (E)> TZEI 129 (D). Strong positive correlations (similarity) were found among the testers B, A, E and D (Figure 1) whereas tester C was found to be unique from all the other testers. Tester C was therefore identified as the most efficient early maturing yellow inbred tester across the four research conditions. Tester B was found to be the most efficient among the testers which were found to be similar, that is A, B D and E. Hence, tester B represented the four testers. Testers C and B were therefore the most efficient among the five testers based on the discriminating ability.

### 3.2 Heterotic grouping of maize inbred lines across research conditions

#### 3.2.1 Heterotic grouping of inbred lines based on HSGCA method

Considering the fact that testers A, B, D and E were similar while tester C was unique, the HSGCA heterotic grouping method classified the 41 inbred lines into three heterotic groups across the research environments (Table 3). Twenty‐two of the inbred lines were placed in heterotic group 1 (heterotic group of testers A, B, D and E), 15 in the heterotic group 2 (heterotic group of tester C) and four in the heterotic group 3 (heterotic group of inbred lines which were not grouped by any of the five testers) (Table 3).

**Table 3:** Classification of early maturing maize inbreds into heterotic groups based on HSGCA effects of grain yield and SNP‐based markers heterotic grouping methods across 17 environments in Nigeria between 2014 and 2016

Group 1 (Testers A, B, D, E)	Group 2 (Tester C)	Group 3 (No Tester)
HSGCA		
TZEI 432,TZEI 441,TZEI 182,TZEI 24,TZEI 175, TZEI 430,TZEI 470,TZEI 472,TZEI 507, TZEI 449,TZEI 450,TZEI 16,TZEI 415,TZEI 428, TZEI 442, TZEI 464,TZEI 508,TZEI 520, TZEI 161,TZEI 173,ENT 8,ENT 17	TZEI 433, TZEI 439, TZEI 443, TZEI 461, TZEI 474, TZEI 483, TZEI 484, TZEI 495, TZEI 515, TZEI 516, TZEI 518, TZEI 522, TZEI 455, TZEI 494, TZEI 160	TZEI 462, TZEI 465, TZEI 486, TZEI 124
SNP‐based markers		
TZEI 16, TZEI 415, TZEI 450, TZEI 173, TZEI 175, TZEI 470, TZEI 472, ENT 17, ENT 8, TZEI 124, TZEI 182	TZEI 160, TZEI 161, TZEI 24	TZEI 428, TZEI 430,TZEI 507, TZEI 474,TZEI 508, TZEI 483, TZEI 484, TZEI 432,TZEI 449, TZEI 486, TZEI 433,TZEI 520,TZEI 443, TZEI 522, TZEI 455, TZEI 515, TZEI 516,TZEI 518, TZEI 439,TZEI 441,TZEI 442,TZEI 461,TZEI 462, TZEI 464,TZEI 465,TZEI 494,TZEI 495

#### 3.2.2 Heterotic grouping of inbred lines based on the SNP marker method

The heterotic grouping method based on the SNP markers also classified the inbred lines into three heterotic groups (Table 3). Group 1 comprised 11 inbred lines whereas group 2 comprised three inbred lines. Group 3 was made up of 27 inbred lines. Only 14 (34%) of the 41 inbred lines were classified similarly into heterotic groups by both the HSGCA and the SNP‐marker‐based heterotic grouping methods.

#### 3.2.3 Comparison of heterotic grouping methods across environments using the breeding efficiency

The HSGCA method identified 68 hybrids as high‐yielding and 28 as low‐yielding across research environments. In contrast, the SNP‐marker‐based method identified 63 hybrids as high‐yielding while 11 were identified as low‐yielding hybrids across research environments (Table 4). The HSGCA method recorded the highest breeding efficiency of 58.1% compared to the 42.2% of the SNP‐marker‐based method (Table 4).

**Table 4:** Number of intergroup and intragroup hybrids classified by the heterotic group's specific combining ability (SCA) and general combining ability (GCA) of grain yield (HSGCA), and the SNP‐based molecular marker methods along with the breeding efficiency (BE) of the methods across 17 contrasting environments in Nigeria between 2014 and 2016

Yield group	Cross type	HSGCA	SNP
1	Inter	68	63
1	Intra	0	5
2	Inter	59	62
2	Intra	9	6
3	Inter	41	58
3	Intra	28	11
BE		58.08	42.21

Note

BE for HSGCA = {[(68/168) × 100) + [(28/37) × 100]}/2; BE for SNP = {[(63/183) × 100) + [(11/22) × 100]}/2.

## 4 DISCUSSION

The significant mean squares of E, G and GEI observed for grain yield and most measured agronomic traits across environments suggested that the test environments were dissimilar and that there were adequate genetic differences among the hybrids for effective selection for the measured traits. The results also revealed that the expression of grain yield and most of the other measured traits were not consistent in the contrasting environments. This result underscores the need for extensive testing of hybrids across multiple locations before release and commercialization.

The failure of the HSGCA and SNP‐marker‐based grouping methods to classify some inbreds into the heterotic groups of the five testers suggested that those inbred lines belonged to heterotic groups other than those of the five testers.

The highest breeding efficiency obtained for the HSGCA heterotic grouping method compared with the SNP‐marker‐based method indicated that the HSGCA method was more effective in classifying the inbred lines into heterotic groups under the contrasting environments. This result confirmed that the HSGCA method was the most reliable for grouping the parental lines into heterotic groups for the development of productive and stable hybrids as well as synthetic varieties. Hence, crossing inbred lines from opposite HSGCA heterotic groups could result in more productive hybrids across drought, low N, *Striga*‐infested and optimal growing environments. Furthermore, the inbred lines classified into the same heterotic group by the HSGCA method could be recombined to form heterotic populations that could be improved through recurrent selection for extraction of inbred lines and synthetics for use in breeding programmes in the tropics. The results obtained in the present study disagreed with those of Badu‐Apraku, Fakorede, et al. (2015), Badu‐Apraku, Annor, et al. (2015), who found the SNP‐based grouping method to be much more efficient for classifying inbred lines under drought, *Striga*‐infested and optimal environments. However, the result is consistent with the findings of Fan et al. (2009), Akinwale et al. (2014), Badu‐Apraku, Fakorede, et al. (2015) and Amegbor et al. (2017) who reported that the HSGCA was the most efficient for classifying inbred lines under drought, low N and optimal environments. The differences observed in the present study and those of the earlier workers could be attributed to genetic differences in the set of inbred lines used in the present study. The classification of the inbred lines into three heterotic groups or clusters (based on the most efficient method, HSGCA) indicated that there was a broad genetic diversity among the set of inbred lines used in the present study.

Another important requirement of any successful breeding programme is the availability of efficient testers which could effectively discriminate and classify inbred lines into appropriate heterotic groups and for the development of high‐yielding hybrids and synthetic varieties. The efficient testers identified in the present study could be utilized for cost‐effective classification of other early‐maturing tropical yellow inbred lines into heterotic groups, assess the combining ability and identify superior hybrid combinations under drought, low N, *Striga,* optimal and across the research environments.

## 5 CONCLUSIONS

The HSGCA method had a higher breeding efficiency than the SNP‐based grouping method across the research environments indicating that it was more effective in classifying the inbred lines into heterotic groups. Maximum heterosis could therefore be exploited if inbred lines with significant and positive GCA effects for grain yield and classified into opposing heterotic groups by the HSGCA method are crossed for hybrid or synthetic variety development. Testers TZEI 17 (B) and TZEI 23 (C) were identified as the most efficient across contrasting environments and would be invaluable for classifying other inbred lines into heterotic groups, assessing the combining ability and developing superior multiple stress tolerant early maturing yellow hybrids for use in the tropics including SSA.

## CONFLICT OF INTEREST

The authors declare that there is no competing interest.

## AUTHORS CONTRIBUTIONS

Benjamin Annor, Baffour Badu‐Apraku and Daniel Nyadanu were involved in the experimental design, data collection, analysis and interpretation as well as write‐up of this research. Richard Akromah and Morakinyo A. B. Fakorede took part in the experimental design, analysis and interpretation of data and writing up.

## Supporting information

Table S1
