# Supplementary material for: Identifying heterotic groups and testers for hybrid development in early maturing yellow maize (Zea mays) for sub‐Saharan Africa
Source: Plant Breed. 2020 Apr 20;139(4):708–16. doi: 10.1111/pbr.12822 (PMC7497213; doi:10.1111/pbr.12822)
Supplement: Supplementary file 1 — Table S1 [file PBR-139-708-s001.doc]

Supplementary Table 1. Environments, locations, research conditions, years, and mean grain yield of early maturing maize hybrids evaluated in Nigeria.

| Environment | Location | Research condition | Year | Grain yield (kg ha^-1^) |
| --- | --- | --- | --- | --- |
| 1 | Minjibir | Managed drought | 2014/2015 | 1782 |
| 2 | Ikenne | Managed drought | 2015/2016 | 1774 |
| 3 | Kadawa | Terminal drought | 2016 | 3360 |
| 4 | Mokwa | Low N | 2015 | 3404 |
| 5 | Ile-Ife | Low N | 2015 | 3220 |
| 6 | Mokwa | Low N | 2016 | 1489 |
| 7 | Mokwa | *Striga* infestation | 2015 | 2602 |
| 8 | Abuja | *Striga* infestation | 2015 | 2815 |
| 9 | Abuja | *Striga* infestation | 2016 | 4426 |
| 10 | Mokwa | *Striga* infestation | 2016 | 2651 |
| 11 | Mokwa | Optimal | 2015 | 6250 |
| 12 | Mokwa | Optimal | 2016 | 3707 |
| 13 | Ikenne | Optimal | 2015 | 4670 |
| 14 | Ikenne | Optimal | 2016 | 4048 |
| 15 | Abuja | Optimal | 2015 | 5208 |
| 16 | Abuja | Optimal | 2016 | 6001 |
| 17 | Ile-Ife | Optimal | 2015 | 4446 |
